# Multivariate meta-analysis of multiple outcomes: characteristics and predictors of borrowing of strength from Cochrane reviews

**DOI:** 10.1186/s13643-022-01999-0

**Published:** 2022-07-26

**Authors:** Miriam Hattle, Danielle L. Burke, Thomas Trikalinos, Christopher H. Schmid, Yong Chen, Dan Jackson, Richard D. Riley

**Affiliations:** 1 Centre for Prognosis Research, School of Medicine, Keele University, Staffordshire, ST5 5BG UK; 2 Department of Biostatistics and Center for Evidence Synthesis in Health, Brown University School of Public Health, Providence, RI 02912 USA; 3 Department of Biostatistics, Epidemiology and Informatics, Perelman School of Medicine, University of Pennsylvania, Philadelphia, PA 19104 USA; 4 Statistical Innovation, AstraZeneca, Academy House, 136 Hills Road, Cambridge, CB2 8PA UK

**Keywords:** Meta-analysis, Borrowing of strength, Multivariate meta-analysis, IPD meta-analysis

## Abstract

**Objectives:**

Multivariate meta-analysis allows the joint synthesis of multiple outcomes accounting for their correlation. This enables borrowing of strength (*BoS*) across outcomes, which may lead to greater efficiency and even different conclusions compared to separate univariate meta-analyses. However, multivariate meta-analysis is complex to apply, so guidance is needed to flag (in advance of analysis) when the approach is most useful.

**Study design and setting:**

We use 43 Cochrane intervention reviews to empirically investigate the characteristics of meta-analysis datasets that are associated with a larger *BoS* statistic (from 0 to 100%) when applying a bivariate meta-analysis of binary outcomes.

**Results:**

Four characteristics were identified as strongly associated with *BoS*: the total number of studies, the number of studies with the outcome of interest, the percentage of studies missing the outcome of interest, and the largest absolute within-study correlation. Using these characteristics, we then develop a model for predicting *BoS* in a new dataset, which is shown to have good performance (an adjusted *R*^2^ of 50%). Applied examples are used to illustrate the use of the *BoS* prediction model.

**Conclusions:**

Cochrane reviewers mainly use univariate meta-analysis methods, but the identified characteristics associated with *BoS* and our subsequent prediction model for *BoS* help to flag when a multivariate meta-analysis may also be beneficial in Cochrane reviews with multiple binary outcomes. Extension to non-Cochrane reviews and other outcome types is still required.

**Supplementary Information:**

The online version contains supplementary material available at 10.1186/s13643-022-01999-0.

## Introduction

Conventional methods for meta-analysis produce a single summary result, for example about one particular outcome. In particular, in most intervention reviews an inverse-variance weighting meta-analysis is typically applied to each outcome of interest separately, and so each meta-analysis utilises just one intervention effect estimate per study. This process can be coined a *univariate meta-analysis*, with the word ‘univariate’ indicating a single summary result is of interest. However, most meta-analysis projects aim to produce multiple summary results, especially because multiple outcomes are of interest, such as a hypertensive participant’s systolic (SBP) and diastolic (DBP) blood pressure, a migraine sufferer’s levels of pain and nausea, a cancer participant’s disease-free and overall survival times, and pregnancy outcomes for both the mother and her baby [1]. This potentially motivates a *multivariate meta-analysis*, to produce multiple summary results (one for each outcome) jointly from the same meta-analysis model [2].

The key advantage of a multivariate meta-analysis of multiple outcomes is to account for their correlation [3]. At the participant level, multiple health outcomes are often correlated with each other, and this leads to correlation amongst multiple effect estimates from the same study. Such correlation of a pair of effect estimates is known as *within-study correlation*. For example, in a randomised trial of anti-hypertensive treatment, the estimated treatment effects for SBP and DBP are likely to have a positive within-study correlation, caused by a positive correlation at the participant level between SBP and DBP. When there is between-study heterogeneity in effects there may also be *between-study correlation,* which arises when the true effect for each outcome is correlated with the true effect for another outcome. For example, the true effect of anti-hypertensive treatment on SBP usually has a positive between-study correlation with the true effect on DBP, caused by changes in study and participant characteristics (such as dose and mean blood pressure at baseline) which modify the true treatment effects on SBP and DBP in the same direction.

Accounting for outcome correlation in a multivariate meta-analysis allows more studies to contribute toward the meta-analysis results for each outcome (i.e. a study can be included in the analysis even though it has not measured all the outcomes of interest), which may improve efficiency and even decrease bias (e.g. due to selective outcome reporting [4]) compared to undertaking a separate univariate meta-analysis for each outcome. In particular, alongside any direct evidence, the multivariate meta-analysis allows the summary result for each outcome to depend on correlated results from other outcomes. The rationale is that by observing information from related outcomes we can learn something about the missing outcomes of interest, and thus gain some knowledge that is otherwise lost; a concept known statistically as *borrowing of strength* (*BoS*) [5, 6]. However, a downside of multivariate meta-analysis is that the approach is more complex than undertaking separate univariate meta-analyses (one for each outcome), as it requires the meta-analyst to obtain or derive within-study correlations between pairs of treatment effect estimates in the same study [7, 8]. Furthermore, the amount of borrowing of strength is often small. Trikalinos et al. examined 45 Cochrane reviews that contained two or three binary outcomes that could be analysed using either univariate or multivariate meta-analysis [9, 10]. They conclude that if the “focus is on the summary effects and the confidence intervals then the choice between the univariate and multivariate meta-analysis has limited practical importance” [9]. Yet, isolated examples within the Trikalinos review do exhibit important differences between univariate and multivariate meta-analysis, and other examples exist where multivariate meta-analysis has an important impact [3, 11–14].

Guidance is therefore needed to help researchers identify (as part of a prespecified analysis and project plan) whether a multivariate meta-analysis may be useful in their particular review. To address this, in this article we use the set of Cochrane reviews identified by Trikalinos et al. to investigate the characteristics of meta-analysis datasets that are associated with larger *BoS* when applying a multivariate meta-analysis. We then derive a multivariable prediction model for predicting the amount of *BoS* conditional on these characteristics, which might be useful as part of a prespecified analysis plan to flag when to use the multivariate approach in practice. Note that we focus on the benefits of multivariate meta-analysis for estimating the summary effect for each outcome, and do not consider functions of the outcomes (e.g. differences in summary effect for outcomes 1 and 2), as then the benefit of a multivariate meta-analysis to account for correlation is more obvious (i.e. to determine the correct variance of the function) [15].

## Methods

We firstly introduce the Trikalinos dataset to be used through the article. Then we describe our methods to identify characteristics associated with *BoS,* and for developing a multivariable prediction model for *BoS*.

### Data analysis using 43 reviews of binary outcomes identified by Trikalinos

There were 45 reviews included in the empirical review by Trikalinos et al. [9, 10]. Each review contained at least seven studies that reported both outcomes or at least half the studies with both outcomes if the total number of studies was greater than 14. Each of the studies satisfying the previous requirement (i.e. at least seven studies with both outcomes) had at least 10 patients and at least two events. There were two reviews that contained three outcomes, but these were excluded for our purposes since we decided to focus on bivariate models. The remaining 43 reviews were included, and these contained between 7 and 132 studies with two binary outcomes of interest each with a cross classification (two by two) table summarising the number of outcome events and non-events for the treatment and control groups. The relationships between the pair of binary outcomes was either mutually exclusive or an is-subset-of relationship. An is-subset-of relationships refers to when one outcome is contained within the other. For example, the number of patients that have survived with a particular condition at, say, 6 months and a year. A mutually exclusive relationship is when the outcomes are independent of each other and therefore occur separately. An example is death from breast cancer and death from other causes, excluding breast cancer.

For each of the 43 meta-analysis datasets, we used the two-by-two tables for each outcome in each trial to derive treatment effect estimates (log odds ratio estimates) and corresponding error variances. A fixed 0.5 continuity correction was required if any denominator in the equation for the variance was equal to zero [16, 17]; that is, if a study had a zero cell in the two-by-two table, then 0.5 was added to all cells for that study. This is a similar approach to the normal approximation analyses in the original Trikalinos review [9, 10]. For a pair of treatment effect estimates in the same trial, we also derived their within-study correlation using the formula provided for an is-subset-of relationship [7, 18], and by Trikalinos and Olkin for a mutually exclusive relationship [19]. In some studies, the within-study correlation was +1 or −1, which can cause issues of singular variance matrices during the multivariate model estimation. To avoid this issue, we replaced any ±1 values with ±0*.*99., although other approaches are possible [18].

To each of the 43 meta-analysis datasets, a univariate common-effect meta-analysis was applied to each outcome separately, using maximum likelihood (ML) estimation. Then we also fitted a bivariate common-effect meta-analysis using ML estimation, to jointly analyse both outcomes whilst accounting for any within-study correlations. The ordering of outcome 1 or 2 was irrelevant (i.e. same results obtained regardless), though for the is-subset-of reviews outcome 2 was designated to be the subset of outcome 1.

A bivariate meta-analysis can be conducted in many statistical packages, for example in Stata and R there is the *mvmeta* package (which, in Stata, also provides an option to calculate the *BoS*), and formula for univariate and bivariate meta-analysis models is shown elsewhere [2, 3].

Following the bivariate analysis, the *BoS* was quantified for each outcome by calculating the *BoS* statistic proposed by Jackson et al. [6]:

$$BoS=100\%\times \left(1-\frac{\mathrm{variance}\ \mathrm{of}\ \mathrm{summary}\ \mathrm{result}\ \mathrm{from}\ \mathrm{multivariate}\ \mathrm{analysis}}{\mathrm{variance}\ \mathrm{of}\ \mathrm{summary}\ \mathrm{result}\ \mathrm{from}\ \mathrm{univariate}\ \mathrm{analysis}}\right).$$

The *BoS* statistic provides the percentage reduction in the variance of a particular summary result that is due to (borrowed from) data from other correlated outcomes. It is the percentage weight toward the summary result for, say, outcome 1 that is given to the study data for other correlated outcomes [6]. For example, in a bivariate meta-analysis, a *BoS* of 0% for outcome 1 indicates that the summary result for outcome 1 depends only on data for outcome 1, i.e., that outcome 2 does not add any information for estimating outcome 1. As *BoS* increases, outcome 2 provides more and more information toward the estimate of outcome 1, reflected by reducing its variance.

The distribution of *BoS* statistic values was summarised using descriptive statistics and graphically via histograms (see Supplementary material).

The process was repeated but rather using univariate and bivariate *random-effects* models, which allow for between-study heterogeneity. Similar conclusions were drawn and so we focus on the results from the common-effect meta-analyses in this paper. Further, some of the bivariate random-effects models suffered from problems estimating the between-study correlation (often ‘converged’ at -1 or +1, for reasons explained elsewhere [20]), and so we deemed it more reliable to focus on *BoS* observed for the bivariate common-effect model. Results from the random-effects models are shown elsewhere [21].

### Examining characteristics associated with *BoS*

The following seven meta-analysis level characteristics were selected for examination of their association with *BoS* statistic values from a bivariate common-effect meta-analysis:

- The percentage of studies with missing data for the outcome of interest
- The percentage of studies with missing data across both outcomes
- The number of studies in the meta-analysis
- The number of studies with only the outcome of interest
- The number of studies with both outcomes
- The average absolute within-study correlation
- The largest absolute within-study correlation

These characteristics were identified by the research team based on analytic reasoning (see Supplementary material), and our previous (applied and methodological) experience [3, 12, 15, 22]. The unadjusted effect of each characteristic on the magnitude of *BoS* was estimated by fitting a linear regression with *BoS* as the outcome and the characteristic as the only covariate. Two *BoS* values were available for each of the 43 reviews (one for each outcome), and so the dataset had 86 outcome values in total. A random intercept was used to account for clustering of *BoS* values from the same study. We also considered modelling *BoS* on the log scale, but this did not change the findings importantly, and therefore we present results on the *BoS* scale to aid interpretation.

### Development and internal validation of a prediction model for *BoS*

A multivariable prediction model was developed for predicting *BoS* in a new bivariate meta-analysis dataset. The 7 characteristics previously listed were candidate predictors for inclusion. A summary of the characteristics of the 43 meta-analyses examined by Trikalinos et al. [9, 10] is provided in Table A in supplementary material. As there were 86 *BoS* values for the modelling, this corresponded to 12.3 values per candidate predictor. At the time of model development, this was considered appropriate as it was larger than ten subjects per predictor (often a rule of thumb for sample size), larger than a recent proposal of two values per predictor [23], and ensured a multiplicative margin of error less than 20% for the residual standard deviation (i.e. lower and upper bounds of 95% confidence for residual variance within 20% of the estimated value) [24, 25].

A multivariable linear regression model containing all the seven candidate predictors (forcing them all to be included, regardless of statistical significance) was fitted. The apparent model performance was quantified by the apparent *R*^2^ statistic. Internal validation was then undertaken to obtain optimism-adjusted estimates of *R*^2^ and calibration slope, using bootstrap resampling with 1000 bootstrap samples, as described elsewhere [26–28]. The optimism-adjusted calibration slope was then used as a uniform shrinkage factor; that is, we multiplied the predictor effects of the fitted model by the optimism-adjusted calibration slope. Then, forcing the revised predictor effects to be held fixed, we re-estimated the model intercept to ensure calibration-in-the-large. This produced our final model with all predictors.

In addition to fitting full models, a backwards selection procedure was undertaken to identify a simpler model, with *p* values less than 0.1 used to define predictor inclusion. Internal validation and optimism adjustment was again applied using bootstrapping, which accounted for the variable selection when estimating optimism.

### Applications in new data

For illustration of their potential use, we applied the developed tools to predict *BoS* in two Cochrane reviews not included in the Trikalinos review, and to three non-Cochrane reviews, with comparison to subsequent multivariate meta-analysis results and observed *BoS* values.

## Results

### *BoS* values and comparison of bivariate and univariate meta-analysis results

**Fig. 1:**
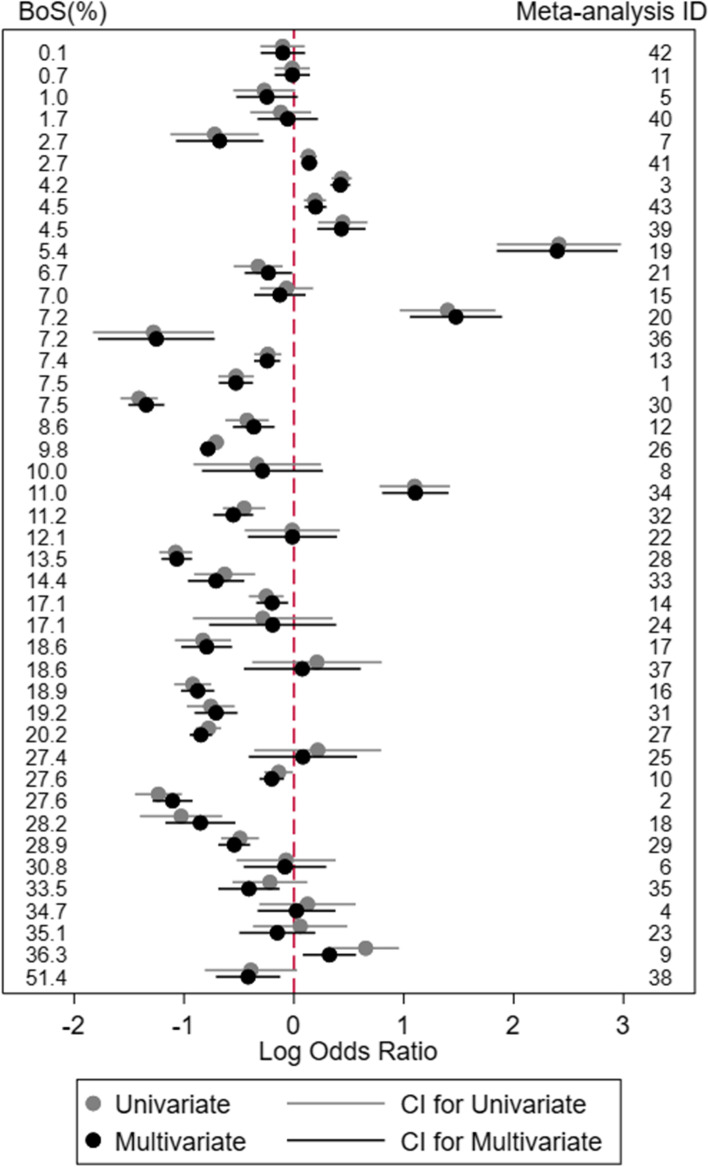
Comparison of the univariate and bivariate meta-analysis results on the log odds ratio scale for outcome 1 from the 43 reviews examined by Trikalinos et al., ordered by the magnitude of the *BoS* statistic

**Fig. 2:**
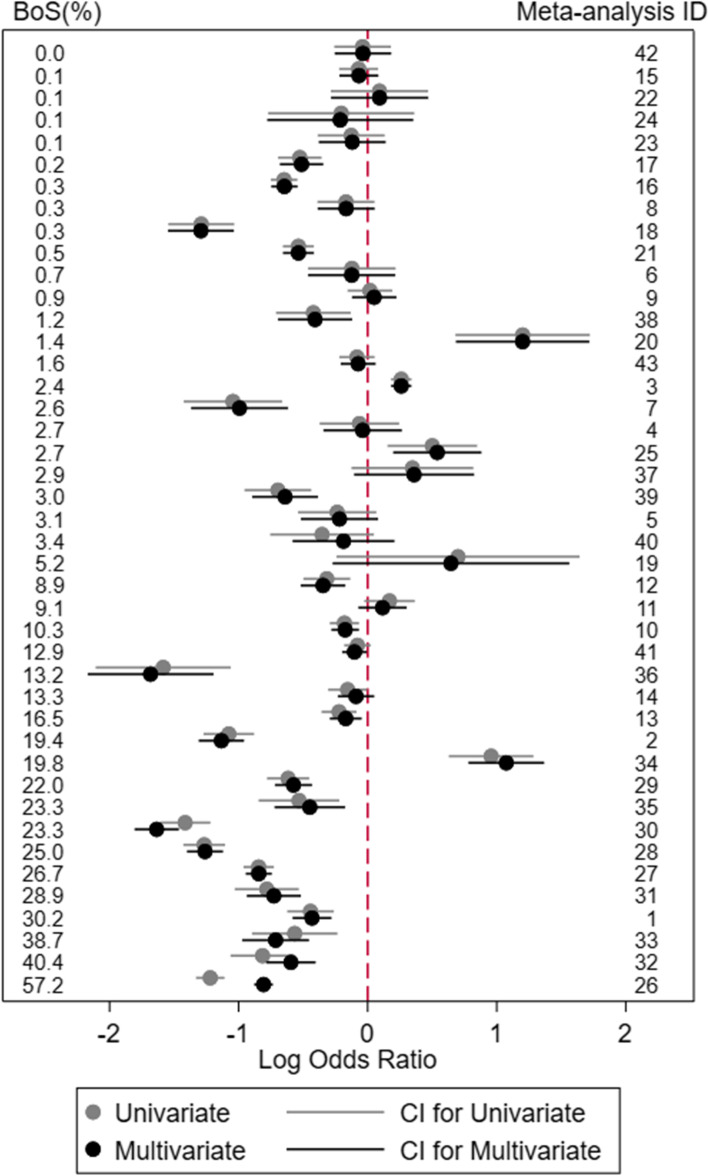
Comparison of the univariate and bivariate meta-analysis results on the log odds ratio scale for outcome 2 for the 43 meta-analyses examined by Trikalinos et al., ordered by the magnitude of the *BoS* statistic

The 86 *BoS* statistic values from the 43 meta-analyses in the Trikalinos review are shown alongside the univariate and bivariate meta-analysis results within Figs. 1 and 2 for outcomes 1 and 2, respectively. (see Fig. A in the supplementary material for outcome 1 and outcome 2 ordered by meta-analysis ID). A large proportion of the *BoS* values were small; the median (mean) value was 9.0% (13.2%), and the inter-quartile range was 2.71% to 20.18%, with a minimum value of 0.05% (Fig. B in the supplementary material). However, 22 of the 86 *BoS* values were over 20%, and the largest value was 57.2%, indicating how a bivariate meta-analysis provides notably greater efficiency than separate univariate meta-analyses in some applications.

Important differences between bivariate and univariate meta-analysis results arise in some examples, and these tend to occur in situations with largest *BoS* values. For example, for outcome 1 in meta-analysis ID 23 (*BoS =* 35.1%), the summary log odds ratio was 0.06 (95% CI: −0.37 to 0.49, odds ratio = 1.06) from the univariate meta-analysis and −0.15 (95% CI: −0.50 to 0.19, odds ratio = 0.86) from the multivariate meta-analysis; hence the direction of the summary effect changed. Similarly, for outcome 1 in meta-analysis ID 9 (BoS = 36.3%), the summary log odds ratio was 0.65 (95% CI: 0.35 to 0.96) from the univariate and 0.32 (95% CI: 0.08 to 0.57) from the multivariate meta-analysis; a difference in the magnitude of the effect size. For outcome 2 in meta-analysis ID 26 (*BoS* = 57.2%), the summary log odds ratio was −1.22 (95% CI: −1.33 to −1.11) from the univariate meta-analysis and −0.81 (95% CI: −0.88 to −0.73) from the multivariate meta-analysis; hence the confidence interval was narrower and the treatment effect less strong after multivariate analysis. Also, for outcome 1 in meta-analysis ID 38 (*BoS =* 51.4%), the summary log odds ratio was −0.39 (95% CI: −0.81 to 0.03; *p* value = 0.068) from the univariate meta-analysis and −0.42 (95% CI: −0.71 to -0.12; *p* value = 0.005) from the multivariate meta-analysis; hence the statistical significance changed. Note that our focus on change in *p* values is merely for illustration, as in practice basing decisions solely on statistical significance is not recommended, and indeed most applications did not result in a change of statistical significance).

### Characteristics associated with *BoS*

Linear regression analyses looking at each predictor individually found strong evidence that all our seven pre-specified characteristics were positively associated with the magnitude of the *BoS* statistic value (Table 1). In particular, the amount of missing outcome data and the magnitude of within-study correlation appeared to be important. There was a 0.49% (95% CI: 0.24%, 0.74%) increase in *BoS* for every 1% increase in the percentage of studies missing an outcome and a 29.08% (95% CI: 16.30%, 41.85%) increase in *BoS* when the largest absolute value of a within-study correlation changed from 0 to 1. This is sensible, as the amount of *BoS* has been shown mathematically to depend on the size of correlation and the amount of missing data [3, 12, 15, 29]. Increasing the number of studies was associated with a small increase in *BoS*.

**Table 1:** Unadjusted association of seven meta-analysis characteristics and the magnitude of *BoS* in a bivariate meta-analysis of two binary outcomes

Characteristic	Unadjusted effect of characteristics on BoS		
	Coefficient	*p* value	95% CI
Number of studies	0.19	<0.001	0.09 to 0.29
Number of studies with outcome of interest	0.17	0.013	0.04 to 0.31
Number of studies with both outcomes	0.36	0.001	0.15 to 0.57
Percentage of studies missing either outcome	0.49	<0.001	0.24 to 0.74
Percentage of studies missing the outcome corresponding to the BoS value	0.52	<0.001	0.38 to 0.67
Average absolute within-study correlation	28.36	<0.001	14.37 to 42.35
Largest absolute within-study correlation	29.08	<0.001	16.30 to 41.85

### Multivariable prediction model for *BoS*

Multivariable modelling results are shown in Table 2. After fitting a full model including all seven characteristics, the apparent proportion of variability explained (*R*^2^) was 0.58. Only four characteristics had strong evidence for an important adjusted association with *BoS* statistic values: the total number of studies, the number of studies with the outcome of interest, the percentage of studies without the outcome of interest, and the largest absolute within-study correlation value in the meta-analysis dataset. These all had a positive adjusted association with *BoS* values, except for the number of studies providing the outcome of interest which had a negative association. The latter makes sense as, after accounting for the total number of studies, a larger number of studies with the outcome of interest leads to less opportunity for borrowing strength from the correlated outcome. After using backwards selection, only these four characteristics were selected (Table 2), and the model performance was very similar to the full model (apparent *R*^2^ = 0.57). For parsimony, we therefore chose to use this reduced model going forward. Bootstrapping identified a small amount of overfitting in the final model after variable selection (optimism-adjusted calibration slope = 0.96; optimism-adjusted *R*^2^ = 0.50). After applying a global shrinkage factor of 0.96 to adjust for overfitting, the final prediction model was (Table 2):

1 $$\begin{array}{c}\mathrm{Predicted}\;BoS\;\mathrm{for}\;\mathrm{outcome}\;\mathrm{of}\;\mathrm{interest}\;(\mathrm{outcome}\;1)\;=\;-13.02\;+\;(0.630\ast\;\mathrm{number}\;\mathrm{of}\;\mathrm{studies}) - (0.755\;\ast\;\mathrm{number}\;\mathrm{of}\;\mathrm{studies}\;\mathrm{with}\;\mathrm{outcome}\;\mathrm{of}\;\mathrm{interest})\;+\;(0.282\;\ast\;\mathrm{percentage}\;\mathrm{of}\;\mathrm{missing}\;\mathrm{data}\;\mathrm{for}\;\mathrm{outcome}\;\mathrm{of}\;\mathrm{interest})\;+\;(25.581\;\ast\;\mathrm{largest}\;\mathrm{absolute}\;\mathrm{within}-\mathrm{study}\;\mathrm{correlation})\\=\;-13.02\;+\;(0.630\ast\;12) - (0.755\;\ast9)\;+\;(0.282\;\ast25)\;+\;(25.581\;\ast0.99)\\=\;20.1\%\end{array}$$

**Table 2:** Adjusted association of seven meta-analysis characteristics and the magnitude of *BoS* in a bivariate meta-analysis

Characteristic	Adjusted effect of characteristic on ***BoS (full model)***	Adjusted effect of characteristic on ***BoS (after backwards selection)***	Final model after applying global shrinkage factor of 0.96
	Coefficient (95% CI) ***p-value***	Coefficient (95% CI) ***p-value***	Coefficient
Number of studies	0.70 (0.13 to 1.27) *0.017*	0.65 (0.19 to 1.12) *0.006*	0.630
Number of studies with outcome of interest	−0.69 (−1.39 to 0.01) *0.052*	−0.79 (−1.38 to −0.19) *0.010*	−0.755
Number of studies with both outcomes	−0.23 (−1.20 to 0.74) *0.64*		
Percentage of studies missing either outcome	−0.12 (−0.53 to 0.28) *0.55*		
Percentage of studies missing the outcome corresponding to the BoS value	0.35 (0.09 to 0.61) *0.009*	0.29 (0.10 to 0.48) *0.003*	0.282
Average absolute within-study correlation	−9.98 (−33.15 to 13.19) *0.39*		
Largest absolute within-study correlation	35.23 (12.49 to 57.98) *0.003*	26.59 (16.53 to 36.65) *<0.001*	25.581
Intercept	−13.90 (−23.22 to −4.58) *0.004*	−14.05 (−22.85 to −5.26) *0.002*	−13.020
**Model performance**	**Apparent** ***R***^**2**^**= 0.58**	**Apparent** ***R***^**2**^**= 0.57**	**Optimism-adjusted*R***^**2**^**= 0.50**

**Fig. 3:**
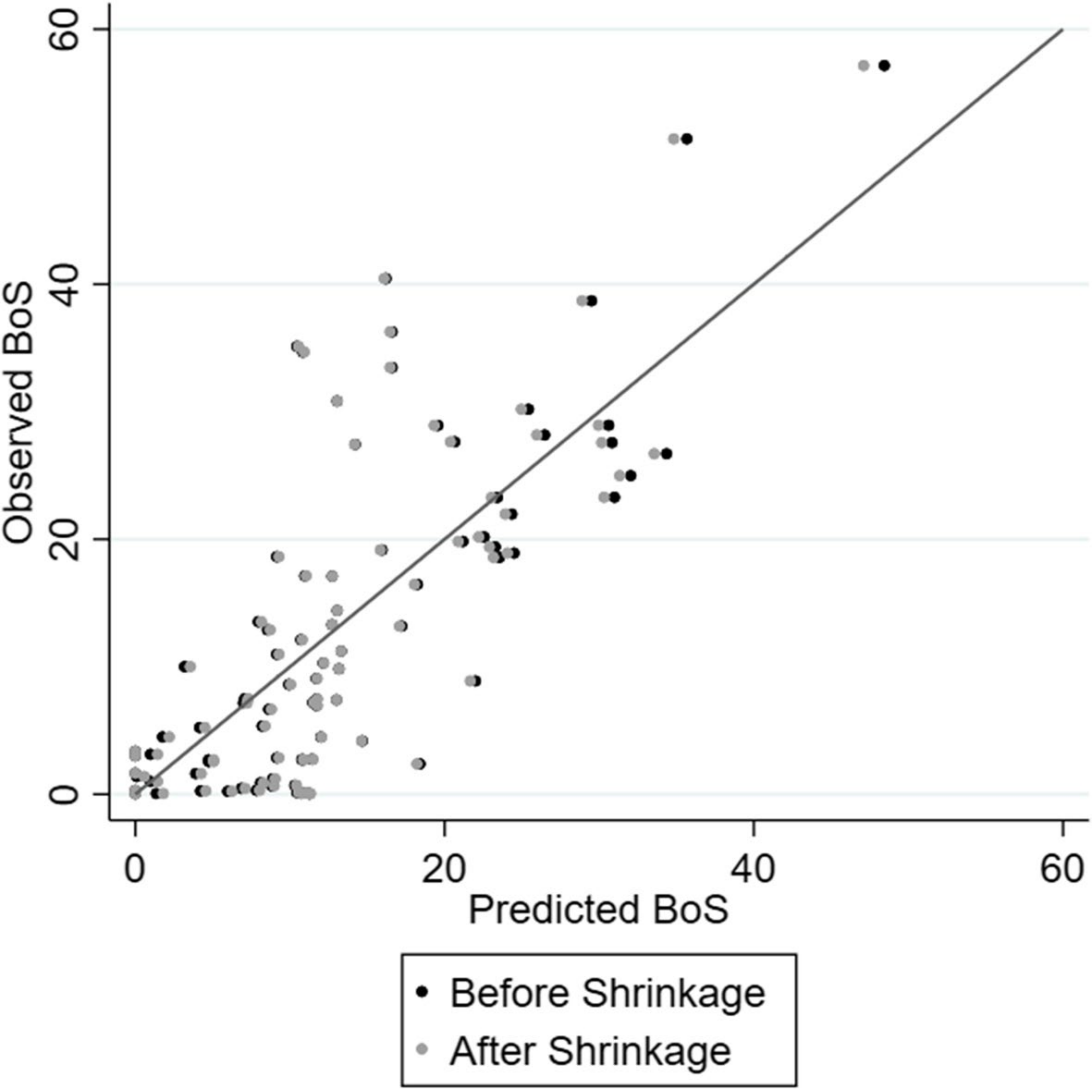
Scatter plot of the observed *BoS* versus the predicted *BoS* from the multivariable model after backwards selection (before and after shrinkage)

A scatter plot of the observed *BoS* values against the predicted *BoS* values (before and after shrinkage) is shown in Fig. 3. The larger the predicted *BoS* then the greater the rationale for undertaking multivariate meta-analysis. We suggest a threshold of about 15 to 20% for flagging that a multivariate meta-analysis is worth considering. Note that the equation depends on knowing the largest within-study correlation. If this is not known, we suggest assuming a large value, say 0.8, for the largest absolute within-study correlation. This would then reveal the predicted *BoS* in a situation where the two outcomes are highly correlated.

#### Applied examples

We applied Eq. (1) to predict *BoS* in two Cochrane reviews published in 2017 by Buzzetti et al. [30] and Feinberg et al. [31], which both contained an is-a-subset-of relationship between two outcomes. Buzzetti et al. reviewed randomised trials comparing the use of glucocorticosteriods for alcoholic hepatitis compared with no intervention, and the two correlated outcomes were mortality at maximal follow-up and mortality at 30 days [30]. Feinberg et al. reviewed randomised trials that compared experimental nutrition support to a control for disease-related malnutrition in Intensive Care Unit participants, including trauma, characterised as “at nutritional risk”; their two correlated outcomes of interest were all-cause mortality at the end of the intervention and at maximum follow-up [31].

The results are shown in Table 3. For the Buzzetti et al. review, using Eq. (1) the predicted *BoS* is only 10.8% for outcome 2, but larger at 20.1% for outcome 1 as calculated below.

$$\begin{array}{c}\mathrm{Predicted}\;BoS\;\mathrm{for}\;\mathrm{outcome}\;\mathrm{of}\;\mathrm{interest}\;(\mathrm{outcome}\;1)\;=\ -13.02\;+\;(0.630\ast\;\mathrm{number}\;\mathrm{of}\;\mathrm{studies})\ -\ (0.755\;\ast\;\mathrm{number}\;\mathrm{of}\;\mathrm{studies}\;\mathrm{with}\;\mathrm{outcome}\;\mathrm{of}\;\mathrm{interest})\;+\;(0.282\;\ast\;\mathrm{percentage}\;\mathrm{of}\;\mathrm{missing}\;\mathrm{data}\;\mathrm{for}\;\mathrm{outcome}\;\mathrm{of}\;\mathrm{interest})\;+\;(25.581\;\ast\;\mathrm{largest}\;\mathrm{absolute}\;\mathrm{within}-\mathrm{study}\;\mathrm{correlation})\\=\ -13.02\;+\;(0.630\ast\;12)\ -\ (0.755\;\ast9)\;+\;(0.282\;\ast25)\;+\;(25.581\;\ast0.99)\\=\;20.1\%\end{array}\\$$

**Table 3:** True and predicted *BoS* values for two Cochrane reviews, alongside their multivariate and univariate meta-analysis results

Review (total number of studies)	Outcome	Number of studies with the outcome	Predicted *BoS* from Eq. (1)	Observed *BoS* after bivariate meta-analysis	Bivariate common-effect meta-analysis		Univariate common-effect meta-analysis for each outcome separately	
					Summary odds ratio (95% CI)	Standard error of log odds ratio	Summary odds ratio (95% CI)	Standard error of log odds ratio
Buzzetti et al. [30] (12)	1: Mortality at 30 days	9	20.1%	47.7%	0.89 (0.69 to 1.15)	0.13	0.69 (0.48 to 0.98)	0.18
	2: Mortality at maximal follow-up	12	10.8%	3.2%	0.89 (0.69 to 1.14)	0.13	0.91 (0.70 to 1.17)	0.13
Feinberg et al. [31] (15)	1: All-cause mortality at end of intervention	11	21.0%	32.8%	0.97 (0.81 to 1.16)	0.09	0.97 (0.78 to 1.20)	0.11
	2: All-cause mortality at maximum follow-up	15	10.4%	0.2%	0.96 (0.82 to 1.12)	0.08	0.96 (0.82 to 1.13)	0.08

Given the predicted *BoS* for outcome 2 is moderately large, it flags that a multivariate meta-analysis may be useful in this review, for outcome 2 to borrow strength from outcome 1. Subsequently, we applied a multivariate meta-analysis and the summary odds ratio for outcome 2 was 0.89 (95% CI: 0.69 to 1.15), which provided much weaker evidence of a beneficial treatment effect than the univariate meta-analysis results (summary odds ratio = 0.69, 95% CI: 0.48 to 0.98). Indeed, the actual observed *BoS* was 47.7% for outcome 2, and so our predicted *BoS* of 20.1% was an underestimate. Nevertheless, the predicted *BoS* still flagged that a multivariate meta-analysis was potentially important. For outcome 1, the observed *BoS* was 3.2%, and so multivariate and univariate meta-analysis results were similar, as anticipated by the lower predicted *BoS* value of 10.8%.

In the Feinberg et al. review, for outcome 1 the predicted *BoS* was 21%, again flagging that a multivariate approach may be worth the extra effort for outcome 1 in this review. When applying the multivariate model, the observed *BoS* was 32.8% for outcome 1, and this led to a narrower confidence interval for the summary treatment effect compared to univariate meta-analysis for outcome 1 (Table 3), although no change in statistical or clinical significance (unlike the Buzzetti et al. review).

## Discussion

### Key findings and recommendations

Methods for multivariate meta-analysis of multiple correlated outcomes are more complex for non-statisticians and may not always be worth the extra effort for reviewers. However, on occasion, the multivariate approach provides important extra efficiency compared to a conventional univariate meta-analysis of each outcome separately and may even lead to different statistical or clinical conclusions. Our empirical examination using the datasets from the Trikalinos review shows that such situations occur mainly when the borrowing of strength amongst outcomes is large. Hence, it is potentially helpful to flag the importance of checking the predicted magnitude of *BoS* to (non-statistical) researchers as part of a pre-specified data analysis plan.

We identified four characteristics as strongly associated with *BoS* in Cochrane intervention reviews with multiple binary outcomes: the total number of studies, the number of studies with the outcome of interest, the percentage of studies missing the outcome of interest, and the largest absolute within-study correlation. Based on these we developed prediction Eq. (1), which can help predict *BoS* for outcomes in a new review. In particular, if this equation predicts *BoS* to be moderate or large (say greater than about 15 to 20%) then it may motivate reviewers to obtain additional statistical support to undertake the multivariate approach and to invest time and resources trying to extract or derive within-study correlations amongst outcomes or even to obtain individual participant data from studies to estimate them directly [32]. It should also be noted that, even if *BoS* is anticipated to be low, sometimes there are other reasons why a multivariate meta-analysis is needed; for example, if functions of summary results are required [9, 10, 12, 15], or if the actual estimate(s) of correlation are of interest.

### Limitations

There are limitations of our work. Although the developed prediction equation explained 50% of the variation in *BoS* values, there can still be a reasonable discrepancy between predicted and observed *Bos* values (see Fig. 3 and Table 3). However, the model is not intended to perfectly predict *BoS* and is rather intended as a tool to provide additional insight for when the multivariate approach is worth considering. This was illustrated in our two applied examples, where outcomes with predicted *BoS* values of > 20% suggested multivariate meta-analysis may be useful, and subsequently applying multivariate meta-analysis improved precision and, in one example, even changed the statistical and clinical conclusions.

Another limitation is that the identified predictors of *BoS* and the developed model are based on Cochrane reviews of binary outcomes, and so further evaluation and extension are needed for other settings, especially continuous outcomes. It is likely that the same predictors will be important contributors of BoS to other settings, although their specific weight in a prediction model may vary.

Also, our prediction model equation requires the researcher to input the largest absolute within-study correlation; this might not be available and in the absence of other information we suggest assuming a large value such as 0.8, to predict *BoS* assuming correlations are high, to see if BoS is likely to be high even in this situation. However, it may be that although the within-study correlations are unknown, a more informed guess can be made based on past research or understanding the clinical outcomes and biology. For example, overall and disease-free survival are likely highly correlated, as are stroke and CVD outcome events, which would justify assuming a large positive correlation. Conversely, benefit and harms might be assumed moderately or highly negatively correlated. Also, IPD could be obtained from a few studies, and the within-study correlations were obtained and used as input values.

The work presented is based on assuming common treatment effects. *BoS* results were also obtained in our examples after using random-effects models to allow for heterogeneity, and similar conclusions found (results shown elsewhere [21]). However, predicting *BoS* in a random effects setting is more complex, due to the impact of between-study variances and between-study correlations, which are difficult to gauge in advance, although informative prior distributions could be considered [33, 34]. Furthermore, *BoS* is harder to define in random effects situations, as the univariate meta-analysis must be forced to have the same between-study variance estimates as the bivariate meta-analysis, to make comparisons fair [6, 22]. Hence, we consider it simpler for researchers to focus on predicting *BoS* initially assuming no heterogeneity.

A further limitation is that the *BoS* statistic is focused on identifying changes in precision from univariate to multivariate meta-analysis; however, in some cases, multivariate meta-analysis may lead to important differences in terms of a change in effect size but not a change in precision, and so the prediction model may not necessarily identify these situations. Thus, choosing to conduct a multivariate meta-analysis over a univariate meta-analysis may depend on other considerations even when BoS is predicted to be small. This requires further research. The feasibility of multivariate meta-analysis should also consider whether within-study correlations and/or IPD can be collected and whether missing outcomes are likely to be missing at random [3, 4].

### Summary

Though multivariate meta-analysis is a more complex evidence synthesis method, it may sometimes be important to consider in reviews of multiple outcomes. We have identified key characteristics and developed a prediction model to help to flag when a multivariate meta-analysis may be beneficial to reviewers with multiple binary outcomes, as part of a pre-specified analysis plan. Extension to other settings, such as non-Cochrane reviews and other continuous outcome types, is still required.

### What this study adds

- Multivariate meta-analysis jointly synthesises multiple correlated effect estimates from multiple studies which enables borrowing of strength across outcomes. This can sometimes lead to difference to results from a standard univariate meta-analysis.
- An empirical examination of Cochrane intervention reviews with multiple binary outcomes shows that multivariate meta-analysis is most influential when the borrowing of strength (*BoS*) amongst outcomes is large.
- Four characteristics were strongly associated with *BoS*: the total number of studies, the number of studies with the outcome of interest, the percentage of studies missing the outcome of interest, and the largest absolute within-study correlation.
- We developed a prediction equation that included these characteristics, to help predict *BoS* for outcomes in a new review, so to flag when the multivariate approach may be worth considering instead of separate univariate analyses.
- We suggest that if this equation predicts *BoS* to be moderate or large (say greater than about 15 to 20%) then a multivariate meta-analysis approach should be considered. However, further evaluation and extension to non-Cochrane reviews and continuous outcomes are still needed.

## Supplementary Information

**Additional file 1.**

## Data Availability

The datasets used and/or analysed during the current study are available from the corresponding author on reasonable request.
