# Supplementary material for: Multivariate meta-analysis of multiple outcomes: characteristics and predictors of borrowing of strength from Cochrane reviews
Source: Syst Rev. 2022 Jul 26;11:149. doi: 10.1186/s13643-022-01999-0 (PMC9316363; doi:10.1186/s13643-022-01999-0)
Supplement: Supplementary file 1 — Additional file 1. [file 13643_2022_1999_MOESM1_ESM.docx]

# Supplementary material

***Within-study correlation formulas***

For the is-a-subset-of relationship the equation for calculating the within-study covariances between the log odds ratios of the outcomes [7, 18]:

$$\sigma_{1,2}=\frac{1}{Nt\left( \frac{xt_{2}}{Nt} \right)\left( 1-\frac{xt_{1}}{Nt} \right)}+\frac{1}{Nc\left( \frac{xc_{2}}{Nc} \right)\left( 1-\frac{xc_{1}}{Nc} \right)}$$

where $xt_{1}$ and $xt_{2}$ are the number of participants in the treatment group with the event for outcomes 1 and 2, respectively; $xc_{1}$ and $xc_{2}$ are the number of participants in the control group, with the event for outcomes 1 and 2 respectively; $Nt$ is the total number of participants in the treatment group; $Nc$ is the total number of participants in the control group.

For the mutually exclusive relationship the equation for calculating the within-study covariances between the log odds ratios of the outcomes [18]:

$$\sigma_{1,2}= -\frac{1}{Nt\left( 1-\frac{xt_{1}}{Nt} \right)\left( 1-\frac{xt_{2}}{Nt} \right)}- \frac{1}{Nc\left( 1-\frac{xc_{1}}{Nc} \right)\left( 1-\frac{xc_{2}}{Nc} \right)}$$

The within-study correlation is then calculated using:

$$\rho_{ws}= \frac{\sigma_{1,2}}{\sqrt{\sigma_{1}^{2}}\sqrt{\sigma_{2}^{2}}}$$

where $\sigma_{1,2}$ is the within-study covariance between the log odds ratios for outcomes; $\sigma_{1}^{2}$ and $\sigma_{2}^{2}$are the variances of the log odds ratio for outcomes 1 and 2, respectively.***Analytic investigation***

We used analytic reasoning to approximate the maximum *BoS*, denoted as max(*BoS*), building on related work by Copas et al.[22] Consider a meta-analysis of *i* = 1 to *N* studies examining a treatment effect on two outcomes. If *M* of the studies were missing outcome 1, then the variance from a univariate meta-analysis would be $var\left( \hat{\theta}_{1} \right)_{N-M}=\left( \sum_{i=1}^{N-M} w_{i} \right)^{-1}$, where $w_{i}=1/(s_{i1}^{2})$ and $s_{i1}^{2}$ is the variance of the treatment effect estimate for outcome 1 in study $i$. Let us assume outcome 2 is available in all $N$ trials and therefore a bivariate meta-analysis could be used to jointly synthesise outcome 1 and outcome 2. In terms of $BoS$ for outcome 1, the maximum gain in information that a bivariate meta-analysis could achieve is to completely recover the lost information for outcome 1. If that were so, the variance of the summary result for outcome 1 from the bivariate meta-analysis would be (because very little borrowing of strength is possible when no data are missing) approximately the same as that from a univariate meta-analysis had all $N$ trials reported outcome 1; i.e. $var\left( \hat{\theta}_{1} \right)_{N}=\left( \sum_{i=1}^{N} w_{i} \right)^{-1}$. This leads to an approximate minimum value of the efficiency, $E$, of $\frac{var\left( \hat{\theta}_{1} \right)_{N}}{var\left( \hat{\theta}_{1} \right)_{N-M}}$ for the bivariate meta-analysis, and therefore

$$\max\left( BoS \right)\approx100\times\left( 1- \left( \min(E \right) \right)\%$$

$$=100\times\left( 1- \left( \frac{var\left( \hat{\theta}_{1} \right)_{N}}{var\left( \hat{\theta}_{1} \right)_{N-M}} \right) \right)\%$$

$$=100\times\left( 1-\frac{\left( \sum_{i=1}^{N-M} w_{i} \right)}{\left( \sum_{i=1}^{N} w_{i} \right)} \right)\%$$

Finally, if all the study weights were the same (i.e. For all $i$, $w_{i}=w)$, then

$$\max\left( BoS \right)\approx100\times\left( 1-\frac{\left( \sum_{i=1}^{N-M} w \right)}{\left( \sum_{i=1}^{N} w \right)} \right)\%$$

$$=100\times\left( 1-\frac{\left( N-M \right)w}{Nw} \right)\%$$

$$=100\times\left( 1-\frac{\left( N-M \right)}{N} \right)\%$$

$$=100\times\left( \frac{M}{N} \right)\%$$

Therefore, we can consider $BoS$ for outcome 1 to be approximately bounded by the percentage of the total studies ($N$) with missing data for outcome 1 ($M$). This might be considered a rule-of-thumb. However, we emphasise this is only an approximation. In truth, $\max\left( BoS \right)$ is more complex than this, as the weights might not be similar in studies with and without missing outcomes (e.g. if there is a systematic difference in their size or number of events); also the spread of weights is important.[22] Indeed borrowing of strength can still occur even when there are no missing outcomes, although will usually be very small.[12, 15]


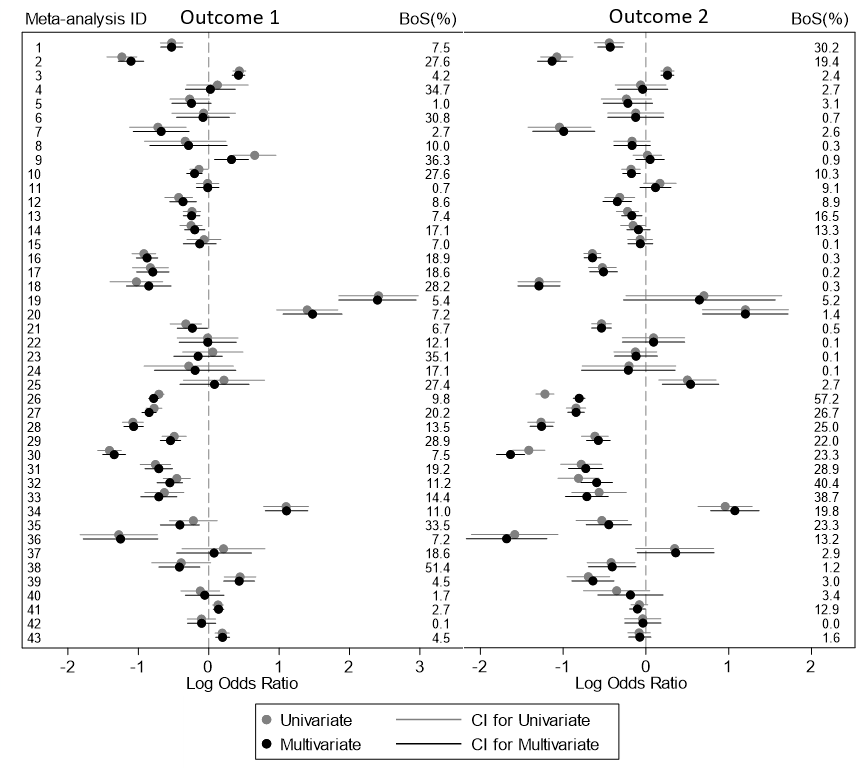
***Comparison of the univariate and bivariate meta-analysis results for outcome one and two for the 43 meta-analyses, ordered by meta-analysis study***

Figure A: Comparison of the univariate and bivariate meta-analysis results on the log odds ratio scale for outcome 1 and 2 for the 43 meta-analysis examined by Trikalinos et al, ordered by meta-analysis ID number

***Histogram of BoS statistic for outcome one and outcome two for the 43 meta-analyses***

Figure B: Histogram of the magnitude of BoS statistic for outcome one and outcome two for the 43 meta-analyses examined by Trikalinos et al.


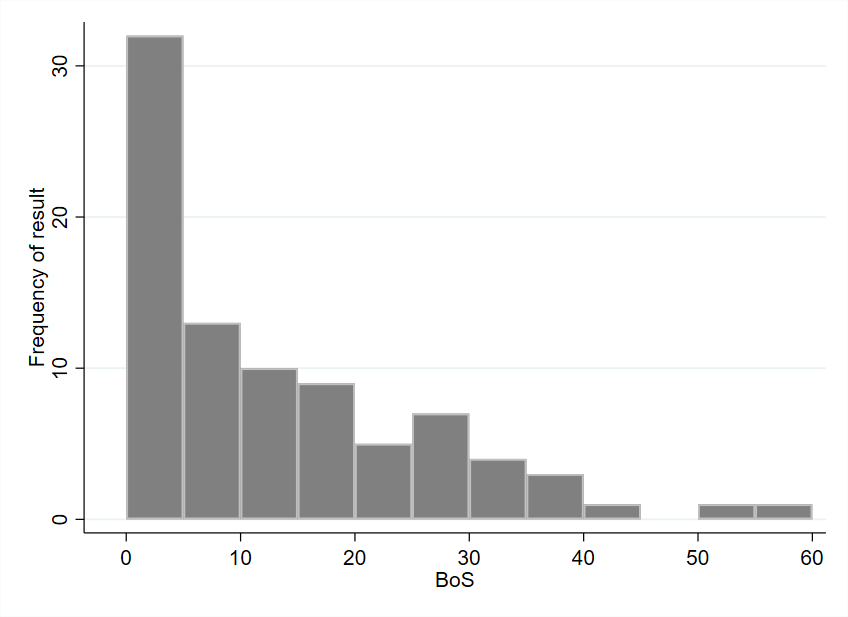


***Table summarising the predictor values of the Trikalinos dataset for the prediction of the BoS statistic***

Table A: Summary of the predictor values for the prediction of the BoS statistic for outcome one and outcome 2 for the 43 meta-analyses examined by Trikalinos et al.

| Meta-analysis ID | Number of studies | Number of studies with O1 | Number of studies with O2 | Number of studies with O1 & O2 | % missing across O1 & O2 | % missing O1 | % missing O2 | Average absolute within-study correlation | Maximum absolute within-study correlation |
| --- | --- | --- | --- | --- | --- | --- | --- | --- | --- |
| 1 | 15 | 14 | 9 | 8 | 23.33 | 6.67 | 40 | 0.82 | 0.94 |
| 2 | 16 | 12 | 11 | 7 | 28.13 | 25 | 31.25 | 0.9 | 0.99 |
| 3 | 10 | 9 | 8 | 7 | 15 | 10 | 20 | 0.64 | 0.99 |
| 4 | 11 | 11 | 11 | 11 | 0 | 0 | 0 | 0.58 | 0.99 |
| 5 | 8 | 8 | 8 | 8 | 0 | 0 | 0 | 0.42 | 0.6 |
| 6 | 15 | 14 | 15 | 14 | 3.33 | 6.67 | 0 | 0.8 | 0.99 |
| 7 | 7 | 7 | 7 | 7 | 0 | 0 | 0 | 0.54 | 0.74 |
| 8 | 8 | 7 | 8 | 7 | 6.25 | 12.5 | 0 | 0.32 | 0.52 |
| 9 | 32 | 27 | 32 | 27 | 7.81 | 15.63 | 0 | 0.56 | 0.99 |
| 10 | 45 | 28 | 41 | 24 | 23.33 | 37.78 | 8.89 | 0.9 | 0.99 |
| 11 | 14 | 14 | 13 | 13 | 3.57 | 0 | 7.14 | 0.63 | 0.93 |
| 12 | 18 | 18 | 13 | 13 | 13.89 | 0 | 27.78 | 0.85 | 0.99 |
| 13 | 16 | 14 | 12 | 10 | 18.75 | 12.5 | 25 | 0.65 | 0.9 |
| 14 | 9 | 8 | 8 | 7 | 11.11 | 11.11 | 11.11 | 0.68 | 0.9 |
| 15 | 11 | 8 | 11 | 8 | 13.64 | 27.27 | 0 | 0.48 | 0.63 |
| 16 | 20 | 11 | 20 | 11 | 22.5 | 45 | 0 | 0.63 | 0.79 |
| 17 | 17 | 10 | 17 | 10 | 20.59 | 41.18 | 0 | 0.63 | 0.84 |
| 18 | 19 | 11 | 19 | 11 | 21.05 | 42.11 | 0 | 0.68 | 0.92 |
| 19 | 9 | 8 | 9 | 8 | 5.56 | 11.11 | 0 | 0.37 | 0.73 |
| 20 | 11 | 9 | 11 | 9 | 9.09 | 18.18 | 0 | 0.42 | 0.59 |
| 21 | 30 | 29 | 30 | 29 | 1.67 | 3.33 | 0 | 0.43 | 0.93 |
| 22 | 12 | 12 | 12 | 12 | 0 | 0 | 0 | 0.85 | 0.99 |
| 23 | 14 | 14 | 14 | 14 | 0 | 0 | 0 | 0.63 | 0.99 |
| 24 | 10 | 10 | 10 | 10 | 0 | 0 | 0 | 0.89 | 0.99 |
| 25 | 11 | 9 | 10 | 8 | 13.64 | 18.18 | 9.09 | 0.68 | 0.86 |
| 26 | 132 | 114 | 79 | 61 | 26.89 | 13.64 | 40.15 | 0.76 | 0.99 |
| 27 | 102 | 80 | 69 | 47 | 26.96 | 21.57 | 32.35 | 0.74 | 0.99 |
| 28 | 70 | 66 | 46 | 42 | 20 | 5.71 | 34.29 | 0.6 | 0.99 |
| 29 | 72 | 58 | 54 | 40 | 22.22 | 19.44 | 25 | 0.72 | 0.99 |
| 30 | 54 | 52 | 34 | 32 | 20.37 | 3.7 | 37.04 | 0.7 | 0.96 |
| 31 | 28 | 24 | 16 | 12 | 28.57 | 14.29 | 42.86 | 0.66 | 0.99 |
| 32 | 14 | 13 | 12 | 11 | 10.71 | 7.14 | 14.29 | 0.68 | 0.99 |
| 33 | 15 | 14 | 8 | 7 | 26.67 | 6.67 | 46.67 | 0.63 | 0.99 |
| 34 | 24 | 24 | 18 | 18 | 12.5 | 0 | 25 | 0.74 | 0.99 |
| 35 | 32 | 27 | 23 | 18 | 21.88 | 15.63 | 28.13 | 0.68 | 0.99 |
| 36 | 14 | 13 | 11 | 10 | 14.29 | 7.14 | 21.43 | 0.72 | 0.92 |
| 37 | 7 | 7 | 7 | 7 | 0 | 0 | 0 | 0.62 | 0.91 |
| 38 | 26 | 12 | 26 | 12 | 26.92 | 53.85 | 0 | 0.89 | 0.99 |
| 39 | 11 | 10 | 11 | 10 | 4.55 | 9.09 | 0 | 0.33 | 0.52 |
| 40 | 10 | 10 | 10 | 10 | 0 | 0 | 0 | 0.37 | 0.55 |
| 41 | 14 | 13 | 14 | 13 | 3.57 | 7.14 | 0 | 0.54 | 0.92 |
| 42 | 31 | 15 | 28 | 12 | 30.65 | 51.61 | 9.68 | 0.03 | 0.06 |
| 43 | 9 | 7 | 9 | 7 | 11.11 | 22.22 | 0 | 0.57 | 0.72 |

O1 = outcome 1, O2 = outcome 2. Further details regarding the Trikalinos et al. dataset can be obtained from Trikalinos et al. [9, 10].
